# Chemical Composition, Antioxidant and Immunomodulatory Properties of Syrah Grape Seed Extract

**DOI:** 10.3390/molecules31040583

**Published:** 2026-02-07

**Authors:** Yavor Ivanov, Zlatina Chengolova, Kalina Tumangelova-Yuzeir, Adelina Yordanova, Ekaterina Ivanova-Todorova, Milena Nikolova-Vlahova, Tzonka Godjevargova

**Affiliations:** 1 Department Biotechnology, Burgas State University “Prof. Dr Assen Zlatarov”, 8010 Burgas, Bulgaria; qvor_burgas@abv.bg (Y.I.); zlatina-chengolova@uniburgas.bg (Z.C.); 2 Laboratory of Clinical Immunology, University Hospital “St. Ivan Rilski”, Department of Clinical Immunology, Medical University of Sofia, 1431 Sofia, Bulgaria; ktuzeir@medfac.mu-sofia.bg (K.T.-Y.); adyordanova@medfac.mu-sofia.bg (A.Y.); etodorova@medfac.mu-sofia.bg (E.I.-T.); 3 Clinic of Nephrology, University Hospital “St. Ivan Rilski”, Department of Internal Medicine, Medical University of Sofia, 1431 Sofia, Bulgaria; mvlahova@medfac.mu-sofia.bg

**Keywords:** grape seed extract, TPC, TF, PC, AO capacity, HPLC analysis, T-helpers type 1, T-helpers type 2, systemic lupus erythematosus

## Abstract

The aim of this study was to obtain and characterize grape seed extract and to demonstrate its potential immunomodulatory properties in the autoimmune disease systemic lupus erythematosus (SLE). A Syrah grape seed extract was obtained using 70% aqueous ethanol, magnetic stirrer, 3 h. The obtained extracts were concentrated by vacuum evaporation and dryer at 40 °C. The total phenolic content (TPC), the total amount of flavonoids (TF) and procyanidins (PC), and the antioxidant (AO) capacity of the extract were determined by spectrophotometric methods. The individual composition of the extract was demonstrated by the high-performance liquid chromatography (HPLC) method. The effect of grape seed extract (GSE) on peripheral blood mononuclear cells (PBMC) from healthy donors and patients with SLE was studied to compare Th1 and Th2 subsets and their expression of key activation markers—CD25 and HLA-DR. PBMC were cultured in the presence or absence of GSE, and the effects on Th1 and Th2 cells were analyzed by flow cytometry. GSE treatment increased the proportion of Th1 cells in both healthy individuals and SLE patients. In addition, a significant upregulation of the late activation marker HLA-DR was observed on Th1 cells obtained from patients with systemic lupus erythematosus (SLE). No significant effects were found on Th2 cell populations. These findings indicate that GSE can stimulate Th1-mediated immune responses in SLE, proving its potential immunomodulatory properties beyond its known antioxidant effects.

## 1. Introduction

The wine industry generates significant amounts of by-products, mainly skins, seeds, stalks [1,2]. The latter represent an important environmental problem, but also an economic challenge, as they contain polyphenols that have valuable biological activities. The highest content of polyphenols is in grape seeds (70%) compared to skins (20%) and stalks (10%) [3]. For this reason, grape seeds are an important by-product for extracting polyphenols from them [4]. Extraction is carried out by conventional and unconventional methods [5,6]. The solid–liquid extraction is one of the most commonly used conventional methods [7,8]. The following solvents are used to conduct this extraction: ethanol, methanol, acetone, diethyl ether, and ethyl acetate mixed with water in different ratios. This method ensures the high yield of extracted phenolic compounds from grape seeds, but used large amounts of organic solvents. The non-conventional methods are supercritical fluid, pressurized liquid, ultrasound-assisted, and microwave-assisted extraction [9,10]. These extraction methods give high extraction degree, significantly reduce the consumption of solvents, and increase the extraction rate.

The most important phenolic compounds contained in grape seed extracts are gallic acid, (−)-epicatechin, (+)-catechin, (−)-epicatechin gallate, procyanidins, etc. [11]. The detection of these compounds is carried out by determining the total phenol content (TPC), total flavonoids (TF) and procyanidins (PC) indicators using spectrophotometric methods [12]. The individual composition of GSE is determined by HPLC or HPLC-MAS [13]. These compounds are strong antioxidants, have antidiabetic, anti-cancer, anti-obesity, anti-inflammatory, antimicrobial, anti-lipid biological effects and are used in medicine, pharmaceuticals, food and beverage industries and cosmetics [14,15,16,17].

A number of publications describe those polyphenols also exhibit immunoregulatory properties, acting on both innate and adaptive immune responses [18]. Experimental studies have shown that grape seed extract can restore the Th1/Th2 balance in mouse models of acute and chronic asthma [19]. A number of pure polyphenolic compounds such as caffeic acid, baicalin and resveratrol have been shown to enhance Th1-type immune responses in infectious diseases. In oncology, multiple studies have shown that polyphenols modulate the tumor microenvironment, restoring Th1 antitumor activity while suppressing Breg, Treg and Th2 populations [20,21,22]. Importantly, polyphenols can exert context-dependent immunomodulatory effects. In disorders characterized by overactive or dysregulated immune responses, polyphenols may exhibit suppressive or regulatory actions. A proanthocyanidin-rich grape seed extract has been reported to exert antiarthritic effects in mice by increasing Th2 and Treg populations, restoring the Th17/Treg balance and reducing the secretion of Th1 cytokines and inflammatory mediators such as MCP-1, MIP-2 and ICAM-1, thereby reducing tissue inflammation [23]. Polyphenols are thought to primarily exert regulatory rather than purely activating effects on the immune system.

Autoimmune diseases (ADs) are a heterogeneous group of more than 80 disorders in which dysregulation of the adaptive immune system leads to immune responses directed against self-antigens [24]. Systemic lupus erythematosus (SLE) is among the most severe ADs, affecting predominantly women aged 15–44 years, with a female-to-male ratio of 9:1 [25,26]. Although the exact etiology of SLE is still not fully understood, it is believed that the disease arises from genome–exosome interactions leading to epigenetic changes and impaired expression of genes implicated in disease pathogenesis [27]. Effector mechanisms leading to activation of autoreactive T-helper (Th) cells are central to the initiation of autoimmune processes. Disturbance of the Th1/Th2 ratio has been implicated in the pathogenesis SLE, with evidence suggesting reduced Th1 percentages or activity in affected individuals [28,29]. Despite the ubiquity of polyphenols in the diets of individuals with SLE, research on their immunomodulatory effects, particularly on major T-helper cell populations, remains limited. Further elucidation of the mechanisms by which GSE influence immune function could provide valuable insights whether they have any therapeutic potential in SLE and other autoimmune disorders.

The main objective of this study was to obtain and characterize a grape seed extract and to investigate its potential immunomodulatory properties in the autoimmune disease systemic lupus erythematosus.

## 2. Results

### 2.1. Chemical Composition of Syrah Grape Seed Extract

#### 2.1.1. Total Phenolic Content, Total Flavonoids, Procyanidins and Antioxidant Capacity of Syrah GSE

The extract is obtained from the seeds of Syrah grape pomace. The seeds are separated from the pomace and dried at 40 °C to constant weight. The harvest is 2025. The vineyards are located in the region of Pomoria, Bulgaria. The extraction is carried out using a water–ethanol solution in a ratio of 30:70, on a magnetic stirrer, for 3 h at 25 °C. The obtained extract is concentrated on a vacuum evaporator to 5 mL. Part of the extract is left in liquid form. The other two parts are dried, one at 40 °C, and the other by lyophilization. The total phenol content (TPC) values of all three forms of extract are determined by Folin–Ciocalteu assay [30]. The results are presented in Table 1.

It is noteworthy that very high TPC values were obtained for all types of extracts. The values range from 226.22 mg GAE/g dw to 240.06 mg GAE/g dw.

All samples were stored at room temperature 23 °C, in a dark place for 6 months. Measurements were made in 0 month and 6 months after the preparation of the three types of extracts. According to the sample physical state, the liquid extract has lower TPC result than the other two types of extracts. The reason about this difference is that the liquid sample is more unstable compared to the other two dried samples. Polyphenols have unsaturated bonds and strong antioxidant capacity, which makes them sensitive to heat, moisture, changes in pH and light [31,32,33,34]. In our case, the liquid sample contains water, which affects the stability of the extract and therefore lower TPC values are measured compared to the other two forms of the extract.

The TPC values of samples dried at 40 °C and lyophilized were very closely. As expected, the lyophilized samples have the higher TPC values, because these samples were dried very well. But overall, all three forms of the extract show high phenolic content.

The total flavonoid content in the studied GSE was also determined using an aluminum complexation assay [35]. Flavonoids include the following compounds: gallic acid, the monomeric flavan-3-ols catechin, epicatechin, gallocatechin, epigallocatechin, epicatechin-3-O-gallate, procyanidin dimers, trimers, and more highly polymerized procyanidins. Table 1 shows that very high values for TF are obtained (from 150.27 to 171.36 mg QE/g dw) in all three forms of the extract, with the liquid extract again showing a slightly lower TF value, for the same reasons as indicated above.

The content of procyanidins in GSE is a very important indicator of its biological activity. Procyanidins are powerful antioxidants that have beneficial effects on a number of diseases. These beneficial compounds are oligomers/polymers of flavan-3-ols. This parameter was determined using the vanillin method [36]. Table 1 shows that the values of procyanidins are very high in its three forms (from 119.88 to 139.14 mg CE/g dw). The extracts lyophilized and dried at 40 °C have slightly higher PC values, as they are more stable.

The antioxidant capacity of the three types of extracts was also determined using ABTS (2,2-azino-bis-3-ethylbenzothiazoline-6-sulphonic acid) and DPPH (1,1-diphenyl-2-picrylhydrazyl) assays (Table 2).

Both methods achieved very high antioxidant capacity values. These results correlate very well with the high TPC, TF and PC values.

As mentioned above, the three forms of GSE were stored in the dark place, at 23 °C for 6 months. The parameters studied above were determined again after 6 months. It was found that all three studied forms of the extract had reduced their values from 6.5 to 8%, while in the lyophilized and dried at 40 °C samples these values had reduced by only 1–2%.

#### 2.1.2. The Individual Compounds in Syrah Seed Extract

**Figure 1:**
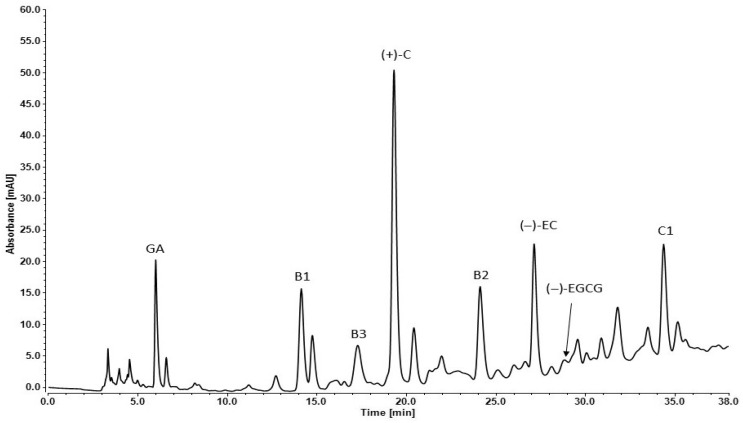
RP-HPLC chromatogram of Syrah seed extract: GA—gallic acid, B1—procyanidin B1, B3—procyanidin B3, (+)-C—(+)-catechin, B2—procyanidin B2, (−)-EC—(−)-epicatechin, (−)-EGCG—(−)-epigallocatechin-3-gallate, C1—procyanidin C1.

The individual compounds of Syrah GSE were determined by reverse phase—high-performance liquid chromatography (RP-HPLC). The analyses were performed by the lyophilized form of the extract. Standard solutions were used for proving the retention times of the grape secondary metabolites. The difference in their affinity to the nonpolar stationary phase (C18) in the column determines their separation. The mobile phase used in that RP-HPLC set was relatively polar at first (90% DI H_2_O), but during the method, its polarity decreased (75% DI H_2_O) (Figure 1).

The retention times (Rt), absorbance (mAU) and concentration (mg/g) of each detected component are presented in Table 3. The seed extracts are rich in monomeric flavan-3-ols: (+) catechin, (−) epicatechin, dimers: procyanidins B1, B2, B3 and trimer: procyanidin C1.

(+)-Catechin had the highest concentration in the analyzed extract compared to the other polyphenols. The other two compounds in high amounts were procyanidin B1 and (−)-epicatechin (Table 3). The chromatogram peaks of gallic acid and procyanidin C1 were similar to the previous two but the exact concentrations were much lower. That effect was due to the different light absorbance capacity of the compounds. Lower absorbance values were obtained as follows: gallic acid, (−)-epigallocatechin gallate, procyanidin B3 and procyanidin C1. The other analyzed plant metabolites had much lower absorbance signal.

### 2.2. Flow-Cytometric Analysis

#### 2.2.1. Gating Strategy

**Figure 2:**
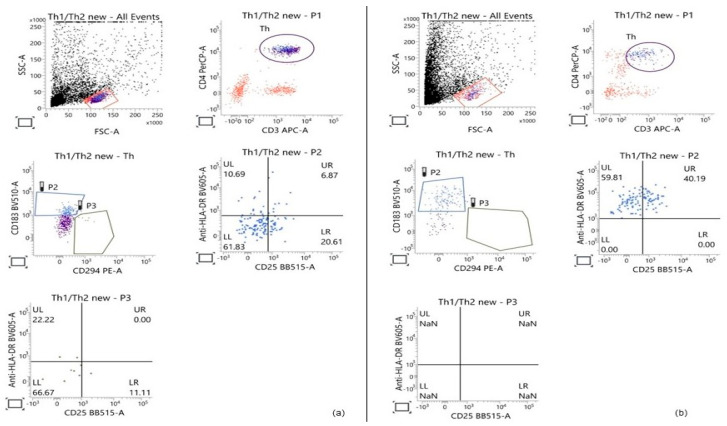
Representative Flow-cytometric analysis showing the gating strategy used to determine the percentage of Th1 and Th2 cells within the T-helper population, based on the expression of the main T-helper markers (CD3 and CD4) and CD183 and CD294, respectively. The percentage of activated cells in each of the two subpopulations was calculated based on the expression of the activation markers HLA-DR and CD25. (**a**) Control PBMCs cultured for 28 h; (**b**) PBMCs treated with 50 μg/mL GSE for 28 h.

Flow-cytometric analysis was based on the initial gating of the lymphocyte population within the PBMC fraction according to cell size and granularity. Subsequently, the population of T-helper cells was identified through the expression of two key T-helper-associated molecules—CD3 and CD4. On a third dot plot, the Th1 and Th2 subpopulations were distinguished based on the surface expression of markers characteristic of each subset: CD183 for Th1 and CD294 for Th2. HLA-DR and CD25 were used to determine the proportion of activated cells within both examined subpopulations. Gating strategy is presented in Figure 2.

#### 2.2.2. Effect of GSE on the Percentage of the Th1 Cell Population

The literature contains extensive evidence indicating that various polyphenolic compounds can skew the immune response toward a Th1-type profile. In patients with SLE, it has been demonstrated that the balance between the two major T-helper subsets, Th1 and Th2, is disrupted in favor of Th2. This information served as the rationale for the present experimental design, with the focus directed precisely toward these two populations.

**Figure 3:**
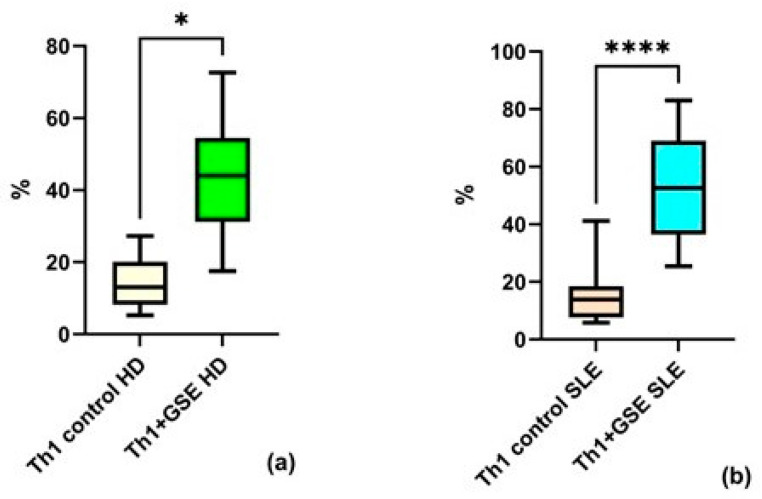
Flow-cytometric analysis of the Th1 subpopulation within the T-helper population: (**a**) a statistically significant increase in Th1 cells cultured with GSE is observed compared with the control cells in healthy donors (HD); (**b**) a statistically significant increase in Th1 cells cultured with GSE is observed compared with the control cells in patients with SLE. In the control PBMC of both groups, the percentage of Th1 cells is comparable. Upon exposure to GSE, this percentage increases to 43.86% ± 17.95 (* *p* = 0.01) in healthy donors and to 52.67% ± 18.62 (**** *p* = 0.0001) in patients with SLE.

Analysis of the obtained flow cytometry data revealed strong agreement with findings reported by other research groups. GSE exerted a pronounced effect on Th1 cells, manifested as a significant increase in the proportion of this subpopulation within the T-helper compartment. This effect was observed both in healthy volunteers (*p* = 0.01) and in patients with SLE (*p* = 0.0001). The percentage of Th1 cells in the T-helpers population of control PBMC samples was similar between the two groups: an average of 14.23% ± 7.64 among healthy donors and 14.63% ± 9.74 among patients with SLE. Following GSE treatment, the Th1 subpopulation increased to an average of 43.86% ± 17.95 in healthy donors (HD), while in SLE patients, the percentage rose to 52.67% ± 18.62 (Figure 3). A stronger effect was observed in the SLE group.

#### 2.2.3. Effect of GSE on the Percentage of the Th2 Cell Population

Analysis of the Th2 subpopulation revealed no significant effect of GSE on the proportion of these cells within the T-helper population. The Th2 percentage in T-helpers of the control PBMC samples was similar between groups: an average value of 7.08% ± 2.66 in healthy donors and 7.17% ± 4.04 in SLE patients.

**Figure 4:**
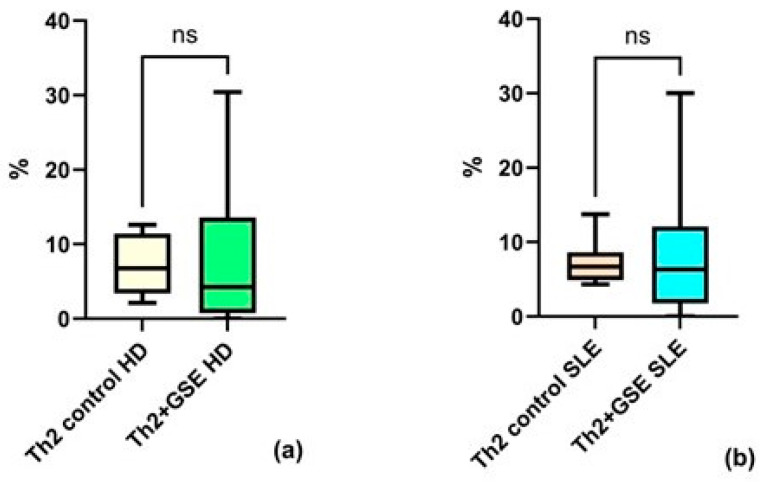
Flow-cytometric analysis of the Th2 subpopulation within the T-helper population: (**a**) no statistically significant (ns) increase in Th2 cells cultured with GSE is observed compared with control cells in healthy donors (HD); (**b**) no statistically significant increase in Th2 cells cultured with GSE is observed compared with control cells in patients with SLE. In the control PBMC of both groups, the percentage of Th2 cells within the T-helper population is similar. Exposure to polyphenols did not result in a significant increase in Th2 cells in either healthy donors (*p* = 0.8438) or SLE patients (*p* = 0.9999).

After GSE exposure, the Th2 subpopulation reached an average of 7.98% ± 11.34 in healthy donors and 8.97% ± 9.39 in patients with SLE (Figure 4). There is no significant effect after the addition of GSE neither for PBMC isolated from HD (*p* = 0.8438), nor for those isolated from SLE patients (*p* = 0.9999).

#### 2.2.4. Effect of GSE on the Activation of the Th1 Cell Population

Flow-cytometric analysis was also conducted to assess the activation status of the two T-cell subpopulations by the expression of CD25 and HLA-DR. CD25 is one of the most important early activation markers appearing rapidly on the T-lymphocyte surface. HLA-DR is a molecule of the Major Histocompatibility Complex (Human Leukocyte Antigen, HLA) and a marker of late activation.

**Figure 5:**
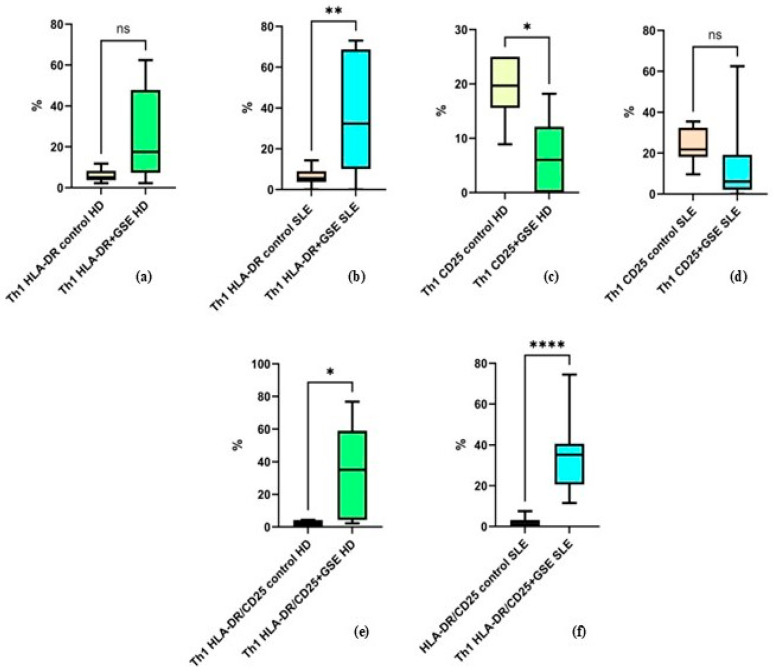
Flow-cytometric analysis of activation marker expression on Th1 cells: (**a**) graph showing the percentage of HLA-DR+ cells in healthy donors (HD) (*p* = 0.074); (**b**) graph showing the percentage of HLA-DR+ cells in SLE patients (** *p* = 0. 0032); (**c**) graph showing the percentage of CD25+ cells in healthy donors (* *p* = 0.0474); (**d**) graph showing the percentage of CD25+ cells in SLE patients (*p* = 0.1704); (**e**) graph showing the percentage of cells double-positive for HLA-DR and CD25 in healthy donors (* *p* = 0.0398); (**f**) graph showing the percentage of cells double-positive for HLA-DR and CD25 in SLE patients (**** *p* < 0.0001).

The results of the analysis of Th1 activation show that GSE exerts a statistically significant effect on HLA-DR expression alone in patients with SLE, whereas no such effect is observed in healthy donors. The percentage of Th1 cells expressing this molecule alone increased from an average of 5.9% ± 3.32 to 25.27% ± 23.34 in healthy donors (*p* = 0.074), and from 6.07% ± 4.27 to 36.93% ± 27.36 in SLE patients (*p* = 0. 0032) following incubation with GSE (Figure 5). Under the influence of GSE, healthy donors exhibited only a trend toward increased expression of this marker.

The percentage of Th1 cells expressing CD25 alone decreased in both groups, from 19.35% ± 5.94 to 6.72% ± 6.88 in healthy donors (*p* = 0.0474) and from 23.84% ± 8.25 to 13.98% ± 18.75 in SLE patients (*p* = 0.1704). This decrease was statistically significant only in healthy donors, while in the SLE group it represented a non-significant trend (Figure 5).

Cells co-expressing both activation markers increased significantly in both groups after GSE incubation, with a stronger effect observed in SLE patients, from 2.11% ± 1.9 to 34.55% ± 28.45 in healthy donors (*p* = 0.0398) and from 1.98% ± 2.35 to 35.43% ± 18.75 in SLE patients (*p* < 0.0001) (Figure 5).

It should be noted that in the control samples, Th1 cells positive for HLA-DR and CD25 individually, as well as the double-positive HLA-DR^+^/CD25^+^ cells, exhibited comparable values between the two groups.

#### 2.2.5. Effect of GSE on the Activation of the Th2 Cell Population

**Figure 6:**
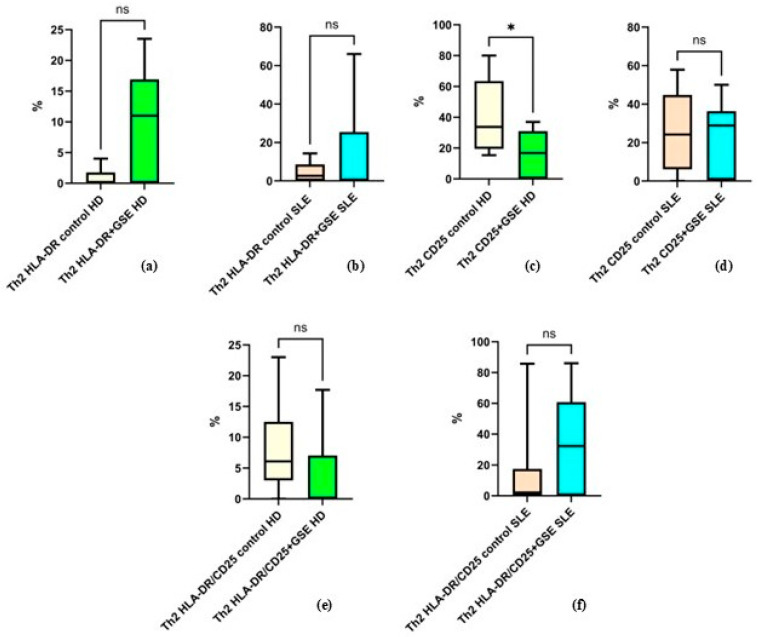
Flow-cytometric analysis of activation marker expression on Th2 cells: (**a**) graph showing the percentage of HLA-DR+ cells in healthy donors (HD) (*p* = 0.1250); (**b**) graph showing the percentage of HLA-DR+ cells in SLE patients (*p* = 0.3008); (**c**) graph showing the percentage of CD25+ cells in healthy donors (* *p* = 0.0105); (**d**) graph showing the percentage of CD25+ cells in SLE patients (*p* = 0.6484); (**e**) graph showing the percentage of cells double-positive for HLA-DR and CD25 in healthy donors (*p* = 0.4375); (**f**) graph showing the percentage of cells double-positive for HLA-DR and CD25 in SLE patients (*p* = 0.0977).

The analysis of Th2 cells and their expression of activation markers did not reveal statistically significant differences following GSE treatment (Figure 6). The only exception was observed for CD25 expression in healthy donors, where GSE exposure resulted in a significant decrease from 40.3% ± 24.73 to 16.67% ± 14.98 (*p* = 0.0105).

The mean values for the other examined groups were as follows: in healthy donors, the percentage of HLA-DR^+^ Th2 cells was 0.8% ± 1.6 in control cells and 10.04% ± 9.04 in GSE-treated cells (*p* = 0.1250); in SLE patients, HLA-DR^+^ Th2 cells accounted for 4.36% ± 5.07 in controls and 14.76% ± 24.80 after GSE treatment (*p* = 0.3008). For CD25^+^ cells in SLE patients, the percentages were 25.39% ± 20.22 in controls and 22.92% ± 19.09 following GSE treatment (*p* = 0.6484). Regarding cells double-positive for CD25/HLA-DR, healthy donors exhibited 8.03% ± 8 in controls and 3.62% ± 7.08 after GSE treatment (*p* = 0.4375), while SLE patients showed 13.59% ± 25.84 in controls and 34.07% ± 30.70 following GSE exposure (*p* = 0.0977).

## 3. Discussion

In recent years, scientific research on the valorization of grape seeds has increased significantly in order to extract and utilize its valuable biologically active substances. These are phenolic compounds, which are secondary plant metabolites, and are present in significant quantities (70%) in grape seeds [3]. These compounds offer potential health benefits and are widely used in the medical, food, nutraceutical, pharmaceutical and cosmetic industries [14,16]. Studies conducted with Syrah GSE convincingly show that the extract is rich in polyphenols. The high values obtained for TPC, TF and PC prove it. The TPC values of liquid, dried at 40 °C, and lyophilized GSE varied from 226.22 mg GAE/g dw to 240.06 mg GAE/g dw. The obtained results are higher than the results presented by other authors. Negro et al. [37] reported that the total phenolic content in red grape seed extracts were 85.80 mg/g dry matter. Guaita et al. [38] described that the values of TPC of seed extracts of four red grape cultivars varied from 73.7 to 107.8 mg GAE/g dw.

The obtained TF results of the three forms of Syrah grape seed extract showed a high content of flavonoids in these extracts. The results varied from 150.27 to 171.36 mg QE/g dw. The obtained values are higher than those cited by Guaita et al. for GSEs (6.90–25.91 mg QE/g dw) [38]. Rockenbach et al. obtained also lower results than our results from 56.01 to 111.21 mg QE/g) [39]. Rajha et al. [40] indicated that the value of total flavonoids of GSEs from Cabernet Sauvignon were 54 mg QE/g dw extract.

The content of procyanidins in GSE is a very important parameter. The obtained results for procyanidins in Syrah GSE varied from 119.88 to 139.14 mg CE/g dw. These results are similar with those obtained by Chengolova et al. [41] for seed extracts of different red grapes (85.2–152.0 mg CE/g dw) and by Nakamura et al. [42]—111 mg CE/g dw.

The HPLC analysis presented that the Syrah seed extracts are rich in monomeric flavan-3-ols: (+)-catechin, (−)-epicatechin, dimers: procyanidins B1, B2, B3 and trimer: procyanidin C1. The concentration of (+)-catechin, (−)-epicatechin and procyanidin B1 are very high. Then followed the concentration of procyanidin B2, procyanidin C and gallic acid. Procyanidin B3 and (−)-epigalocatechin-3-gallate have lower concentrations. Escribano-Bailon et al. [43] reported that the individual compounds in grape seeds of *V. vinifera* (Tintal del pais), according to their concentration, were arranged in the following order: (+)-catechin (11%), (−)-epicatechin (10%), (−)-epicatechin-3-O-gallate (9%), procyanidin B1 (7%). Rodríguez Montealegre et al. [44] determined, by HPLC analysis, that grape seeds contained catechin, epicatechin, epicatechin gallate, protocatechuic acid, procyanidin B1, procyanidin B2, procyanidin B3 and procyanidin B4 as well as gallic acid.

A substantial body of scientific literature indicates that various polyphenolic compounds can redirect the immune response toward a Th1-type profile in conditions with a Th2 predominance. In patients with systemic lupus erythematosus, the balance between the two major T-helper populations, Th1 and Th2, is known to be disrupted, with a shift toward Th2 dominance [28]. This observation provided the rationale for the present experimental design, which focused specifically on these two T-helper subsets. The obtained results align with existing evidence regarding the effects of polyphenols on T-helper subpopulations in various non-autoimmune conditions, including allergic, malignant, and infectious diseases [22]. Regardless of the small sample number included, grape seed extract exhibited a pronounced effect on Th1 cells, reflected in a significant increase in the proportion of this subpopulation within the overall T-helper compartment in both groups investigated (Figure 3). The large standard deviation observed in baseline Th1 results may be attributable to the limited probes number and/or to differences in individual immune responses.

Only one study examining the impact of GSE on T-helper cells was identified, conducted exclusively in healthy donors [45]. Its findings correspond to those of the present work, reporting increased expression and secretion of the primary Th1 cytokine IFN-γ. Other studies regarding proanthocyanidins, one of the main bioactive constituents of GSE, have shown the modulation of T-cell-mediated immune responses. In accordance with our experimental findings, their involvement in Th1 immune polarization has been reported. For example, Sung NY et al. concluded that procyanidin trimer C1 induces macrophage activation via the NF-κB and MAPK pathways, leading to TNF-α and IL-12 secretion and promoting T-helper cell polarization toward a Th1 phenotype [46]. Similarly, a study by Vaid M. et al. in a murine model further supports an indirect modulation of the Th1 immune response through IL-12 induction, reporting a very strong (5- to 8-fold) increase in Th1 cytokine secretion by CD8^+^ T cells and a concomitant reduction in Th2 cytokine secretion by CD4^+^ T cells isolated from mice treated with grape seed proanthocyanidins, compared with untreated controls [47]. These results are consistent with our findings for a substantial elevation of Th1 subpopulation proportion in heathy donors (baseline average 14.23% ± 7.64 increase following GSE treatment to an average of 43.86% ± 17.95) and patients with SLE (baseline average 14.63% ± 9.74 increase following GSE treatment to 52.67% ± 18.62).

A comparative analysis of GSE effects on Th1 and Th2 in both study groups is presented in Figure 3 and Figure 4. The results show similar baseline Th1 and Th2 percentages in PBMCs from healthy donors and SLE patients. The observed results are most likely explained by patients’ clinical characteristics and treatment regimens. Patients included in our cohort showed no detectable clinical or immunological disease. This finding is in line with Dolff et al. [28] who reported no difference in spontaneous expression of IFN-γ, IL-4 and IL17A by T-helpers between SLE patients and healthy controls.

No prior reports were identified addressing the expression of the T-cell activation markers CD25 and HLA-DR following exposure to GSE. CD25, the α-chain of the trimeric IL-2 receptor, is a key marker of early T-cell activation, upregulated within 24 h of TCR stimulation and remaining elevated for several days [48]. HLA-DR, a MHC class II molecule, is constitutively expressed on B cells, monocytes, and macrophages and appears during late activation stages on T and NK cells, serving as a late activation marker [49]. Our results demonstrate a strong effect of GSE on Th1 cells, leading to an increased percentage of cells expressing HLA-DR alone in SLE patients and those co-expressing HLA-DR and CD25 in both study groups, with a more pronounced effect in PBMCs from SLE patients. In contrast in HD, GSE decreased the proportion of cells expressing CD25 alone and had no effect on HLA-DR expression (Figure 5). This result can be attributed to the regulatory effect of GSE on different type of immune systems—dysregulated or well balanced. Control PBMCs from both groups showed similar baseline expression of activation markers, highlighting the stimulatory effect of GSE on Th1 cells and its capacity to promote both the proliferation and terminal differentiation of this subpopulation.

Analysis of the proportion and activation markers in Th2 cells was consistent with previous reports indicating that, under conditions favoring a Th2 response, polyphenols enhance Th1-mediated immunity [22]. Thus, the proanthocyanidin-rich extract exhibited no measurable effect on Th2 frequency or activation status (Figure 4 and Figure 6). The only significant finding—a reduction of CD25 expression in healthy donors—suggests a potential suppressive or regulatory effect of GSE under conditions of a well-regulated immune system.

As mentioned, polyphenols are believed to exert regulatory functions within the human body. Based on current evidence, it may be hypothesized that the stronger effect of GSE on Th1 cells isolated from SLE patients reflects the influence of these compounds on the disrupted immune balance characteristic of the disease. Women typically exhibit a predominance of humoral, or Th2-type, immunity characterized by enhanced antibody production. SLE predominantly affects women of reproductive age, and strong associations have been established between the disease and enhanced humoral immunity. This is reflected in the production of autoantibodies that contribute to SLE pathogenesis [25,50]. Restoring the balance between T-helper subpopulations may thus have a beneficial effect on the dysregulated immune system in SLE.

The application of GSE in both studied groups causes an increase in standard deviation in some of the results, indicating a differential effect of the extract on a subset of the analyzed samples. This observation may be attributed to the limited sample number, as well as to interindividual differences in genetic background and the immune response mechanisms involved.

It is important to emphasize that the present experiments were conducted in vitro and thus reflect only the direct interaction between the extract and the analyzed cell populations. Despite the observed data, it remains premature to conclude whether GSE can exert a clinically meaningful immunoregulatory effect in SLE patients, particularly considering the reduced number and/or functionality of Th1 cells described in this disease. In vivo application may produce different outcomes due to the complexity of physiological processes in both healthy persons and SLE patients. Additional animal models’ studies and human trials are required to evaluate the safety of GSE in SLE patients and to validate the effects observed. Nevertheless, such in vitro studies provide essential insights into the mechanistic effects of GSE on key lymphocyte populations within PBMCs.

### Limitations

The main limitation of this study is the small number of included SLE patients and healthy donors, namely 12 and 6, respectively. This is an initial investigation of the effects of GSE on two main T-cell subpopulations in SLE patients. In this manuscript, we report the results without claiming their clinical relevance, particularly considering the complex immunological mechanisms involved in the pathogenesis of SLE. Additional in vitro and in vivo studies are required to confirm the effects of GSE observed here.

## 4. Materials and Methods

### 4.1. Materials

The grape seed extracts used in this study were from the vinification of red wine *Vitis vinifera* L. cv. Syrah, grown in the Pomorie region, Bulgaria.

The ethanol 99.9% *v*/*v* was from Valerus, Sofia, Bulgaria. Acetonitrile, methanol, phosphoric acid, sodium carbonate, sodium nitrate, aluminum trichloride, sodium hydroxide, hydrogen chloride, Folin–Ciocalteu reagent, vanillin, 2,2-diphenyl-1-picrylhydrazyl, 2,2′-azino-bis(3-ethylbenzothiazoline-6-sulfonic acid), potassium persulfate, gallic acid, (+)-catechin, (−)-epicatechin, (−)-epigallocatechin gallate, procyanidin B1, procyanidin B2, procyanidin B3, and procyanidin C1 were purchased from Sigma-Aldrich Co., Steinheim am Albuch, Germany. The used water was deionized by ELGA’s water purification systems (High Wycombe, UK). The reagents for HPLC analyses were reference standard grade.

For isolation and cultivation of PBMC, the following materials were used: BD Vacutainers CPT; NH: 130 IU, Ficoll (BD, Franklin Lakes, NJ, USA); Cell Culture Plate (Biologix Europe GmbH, Hallbergmoos, Germany), RPMI 1640 Medium (Capricorn scientific GmbH, Ebsdorfergrund, Germany); fetal bovine serum (FBS) (PAA Laboratories, Pasching, Austria); antibiotics/antimycotics (PAA Laboratories, Pasching, Austria).

For the Flow-cytometric analysis: WASH solution (BD, Franklin Lakes, NJ, USA); monoclonal antibodies: anti-CD3-APC, anti-CD4-PerCP, anti-CD294-PE, anti-CD183-BV510, anti-CD25-BB515, anti-HLA-DR-BV605, and cell staining buffer for immunofluorescence (all from BD, Franklin Lakes, NJ, USA); CellFix (BD, Franklin Lakes, NJ, USA).

### 4.2. Preparation of Grape Extract

Grape pomace from Syrah was used for seed obtaining. The optimization of the procedure of grape extract obtaining was discussed in our previous study [51]. The method used in this study is described below.

The grape seeds were washed carefully in water and then dried with oven (Heraeus acutherm, Hanau, Germany) at 40 °C for 24 h. After that, a grinder was used for smaller particle obtaining. The extraction procedure started with 70% ethanol in DI water added in ratio 10 g seed particles in 100 mL organic solvent. The suspension was stirred 3 h, at 500 rpm, room temperature with a magnetic stirrer (MMS-3000, Boeco, Hamburg, Germany). The extracted polyphenols were in the liquid phase, so a centrifugation was made for clean extract obtaining. The conditions were 6000 rpm, 10 min in a centrifuge CompactStar CS 4, VWWR, Equipnet, Boston, MA, USA. The supernatant was concentrated by a vacuum evaporator (Rotavapor R-215, Buchi, Flawil, Switzerland) at 45–50 °C and 100–175 hPa. The final volume of the concentrated extract was 5 mL and it was divided in three glasses. Glass 1 was the obtained liquid extract, glass 2 was extract dried at 40 °C, and glass 3 was lyophilized (VirTis, Los Angelis, CA, USA) (initial temperature between −40 °C and −50 °C, sublimation up to 0 °C with 10^3^ torr, positive temperature step to 40 °C and 10^2^ torr, for 4–8 h). All the obtained samples were stored in a dark place at 23 °C.

### 4.3. Determination of Total Phenolic Content of GSEs

The tree types of GSEs (liquid, dried at 40 °C, lyophilized) were used for TPC. First, the liquid GSE (200 μg/mL) 10 μL was diluted with 990 μL ethanol. The obtained reaction mixture was homogenized with a vortex. The other two types of GSE (dried at 40 °C and lyophilized) were prepared in separate vials and weighted 2 mg each. Then, they were dissolved in ethanol/water in ratio 7/3 with final volume 1 mL each and vortexed. After that, the three types GSE were centrifuged (13,000 rpm, 15 min). The supernatants were used for color reaction with Folin–Ciocalteu [30].

Each sample (50 μL) was mixed with 2 N Folin–Ciocalteu reagent (50 μL) and 75 g/L sodium carbonate (300 μL). The color appeared for 30 min in dark. Finally, the color reaction mixtures were diluted with 2.6 mL DI water each and the absorbance was read at 725 nm with a 6900 UV-Vis JENWAY spectrophotometer (Colmworth, UK). The color reaction was made with standard solutions of gallic acid (50–450 μg/mL in ethanol) for a calibration curve to be obtained. All the samples were tested in triplicate. TPC was calculated as milligram-equivalent gallic acid per gram of dried weight (mg GAE/g dw).

### 4.4. Determination of Total Flavonoids of GSEs

The initial preparation of the tree types GSEs was as mentioned above. The difference is only in the used first concentration of the liquid GSE: 10 μL with concentration 0.8 mg/mL. The other steps of vortex mixing and centrifugation were the same. The TF was determined of the supernatants of the three types GSEs using an aluminum complexation assay [35].

The supernatant (100 μL) was diluted with 1 mL DI water and 5% NaNO_3_ (75 μL) was added. The mixture reacted for 5 min, followed by 6 min reaction with 2% AlCl_3_ (150 μL). Then, DI water (4 mL) and 1N NaOH (500 μL) were added and the mixture reacted 11 min in dark. The absorbance was read at 510 nm on the 6900 UV-Vis JENWAY spectrophotometers (Colmworth, UK). The color reaction was made with quercetin (0.1–1 mg/mL in methanol) for a calibration curve obtaining. All the samples were tested in triplicate. TF were expressed as milligram-equivalent quercetin per gram of dry weight (mg QE/g dw).

### 4.5. Determination of Procyanidins of GSEs

PC were measured by Brezoiu et al. [36] procedure by initial dilution of the three types GSEs in methanol to concentrations 300 μg/mL. The sample mixture contained GSE (1 mL), 1% vanillin (2.5 mL) and 9 mol/L HCl (2.5 mL). A control mixture was prepared too, containing GSE (1 mL), methanol (2.5 mL) and 9 mol/L HCl (2.5 mL). Correction of the signal was made with blank mixture, containing methanol (1 mL), 1% vanillin (2.5 mL) and 9 mol/L HCl (2.5 mL), and zero mixture, containing methanol (1 mL), methanol (2.5 mL) and 9 mol/L HCl (2.5 mL). The mixtures reacted 20 min, 30 °C and absorbance of was read at 500 nm using the JENWAY 6900 spectrophotometer (Colmworth, UK).

The method was also made with standard solutions of (+)-catechin (20–500 μg/mL) for calibration curve obtaining. All the samples were tested in triplicate.

The correction of the absorbance signal was made by the following equation:

A = (A_s_ − A_b_) − (A_c_ − A_0_)

A—corrected absorbance,

A_s_—absorbance of the sample mixture,

A_b_—absorbance of the blank mixture,

A_c_—absorbance of the control mixture,

A_0_—absorbance of the zero mixture.

The PC in each test solution was calculated from the calibration curve and expressed as mg (+)-catechin per gram of dried weight (mg CE/g dw).

### 4.6. Determination of Antioxidant Capacity by ABTS Assay

The ABTS analysis started with ABTS radical (ABTS+●) obtaining by mixing of equimolar amounts of 2.6 mM of potassium persulfate and 7.4 mM ABTS, incubated 16 h in dark. The obtained ABTS+● was diluted with methanol (60 mL) to absorbance 1.1 ± 0.02 at 734 nm [52]. Then the samples were prepared, containing GSE (1 mg/mL in methanol, 100 μL) and ABTS+● (2 mL), and incubated 10 min, in dark. Absorbance was read at 734 nm on the UV-Vis JENWAY 6900 spectrophotometer (Colmworth, UK). All the samples were tested in triplicate. The inhibition of the ABTS+● activity is presented in per cent by following equation:

% Inhibition of ABTS+● activity = (A − B)/A × 100

A—absorbance of the ABTS+● solution before the GSE addition,

B—absorbance of the sample mixture.

### 4.7. Determination of Antioxidant Capacity by DPPH Assay

The antioxidant activity was measured by DPPH• method [52]. The GSE (1 mg/mL in methanol, 100 μL) and fresh prepared DPPH (24 mg in 100 mL methanol, 2 mL) were mixed and incubated 20 min. The absorbance was read at 515 nm on the UV-Vis JENWAY 6900 spectrophotometer (Colmworth, UK). All the samples were tested in triplicate. The inhibition of the DPPH● activity is presented in percentage by following equation:

% Inhibition of DPPH● activity = (A − B)/A × 100

A—absorbance of the DPPH● solution before the GSE addition;

B—absorbance of the sample mixture.

### 4.8. RP-HPLC Analyses of Grape Seed Extracts

The RP-HPLC analyses were performed by Dionex UltiMate 3000 UHPLC with a diode array detector (Thermo Fischer Scientific, Waltham, MA, USA) set at 280 nm, and a column Agilent Zorbax Stable bond C18 (250 × 4.6 mm ID, 5 μm) with a guard column Zorbax Stable bond C18 (12.5 × 4.6 mm ID, 5 μm). The analyses were at 24 °C in the column oven. The flow rate was 0.7 mL/min. The sample loaded in the column was 10 μL Syrah seed powder dissolved in 90% DI water with 0.1% phosphoric acid and 10% acetonitrile. The RP-HPLC gradient of the mobile phase was made by initial 90% DI water with 0.1% phosphoric acid and 10% acetonitrile for 7 min. After that, the acetonitrile concentration was increased to 20% for 33 min. Finally, the acetonitrile concentration reached 25% for another 25 min. The eluents and the sample were filtered by 0.45 μm filter units.

The obtained peaks in the chromatograms were proved by standard solution of the polyphenolic compounds: gallic acid, (+)-catechin, (−)-epicatechin, (−)-epigallocatechin gallate, procyanidin B1, procyanidin B2, procyanidin B3, and procyanidin C1. The standards were used for trendline obtaining and derivation of equations and R^2^.

### 4.9. Blood Samples Collection

For the purposes of the present study, blood samples were obtained from 12 patients diagnosed with systemic lupus erythematosus (SLE) at the Nephrology Clinic of UH “St. Ivan Rilski”—Sofia, and from 6 healthy volunteers. All samples were collected in accordance with Good Laboratory Practice (GLP) standards and were immediately transported for subsequent processing at the Clinical Immunology Laboratory of the same medical institution. The mean age of the patients was 59.6 years (range: 30–88 years), with a male-to-female ratio of 5:7. The mean age of the healthy controls was 49 years (range: 27–73 years), with a male-to-female ratio of 2:4. Peripheral blood samples from patients with systemic lupus erythematosus (SLE) were obtained at times of no detectable clinical or immunological disease activity. Clinical, immunological, and demographic data of the study cohort are detailed in Table 4.

### 4.10. Isolation and Cultivation of PBMC

Peripheral blood mononuclear cells (PBMC) from patients with SLE and healthy volunteers were isolated from peripheral blood samples using a Ficoll density gradient and centrifugation. For this purpose, specialized collection tubes—BD Vacutainers CPT; NH: 130 IU, Ficoll: 2 mL (BD, USA)—were used. According to the manufacturer’s instructions, the tubes were centrifuged for 10 min at 1600× *g* (Universal 320 R, Hettich, Germany). Subsequently, PBMC were sterile isolated under a laminar flow hood (Lamin Air HLB 2448, Heraeus, Germany) and transferred into 15 mL tubes for subsequent washing with 10 mL of physiological saline solution and centrifugation at 300× *g* for 10 min. The supernatant was discarded, and PBMC were resuspended to a concentration of 2–3 × 10^6^ cells/mL. One milliliter of the prepared cell suspension was seeded into 6-well culture plates (Cell Culture Plate, Biologic Europe, Germany), followed by the addition of 2 mL of culture medium containing RPMI 1640 Medium (Capricorn scientific GmbH, Germany), 10% (*v*/*v*) fetal bovine serum (FBS) (PAA Laboratories, Austria), and antibiotics/antimycotics at 100 IU/L (PAA Laboratories, Austria). In each well, the PBMC concentration was maintained at 2–3 × 10^6^ cells.

The PBMC were seeded for 28 h in two wells—one control well (only with medium) and one for culturing with GSE. The cells were treated with GSE twice at 0 h and the 24 h. After 28 h, the PBMC from both wells were collected, centrifuged for 10 min at 300× *g*, and prepared for Flow-cytometric analysis.

### 4.11. Flow-Cytometric Analysis

Flow-cytometric analysis was performed according to the standard surface staining protocol provided by the manufacturer of the monoclonal antibodies. PBMC from each well (treated with extract and control) were washed with 2 mL of WASH solution (BD, Franklin Lakes, NJ, USA) and centrifuged at 300× *g* for 10 min. The supernatant was discarded, and cells were stained with the following antibodies: anti-CD3-APC, anti-CD4-PerCP, anti-CD294-PE, anti-CD183-BV510, anti-CD25-BB515, anti-HLA-DR-BV605, and cell staining buffer for immunofluorescence (all from BD, Franklin Lakes, NJ, USA). After 30 min of incubation, cells were washed again with 2 mL of WASH solution and centrifuged at 300× *g* for 5 min. Following removal of the supernatant, cells were fixed with CellFix (BD, Franklin Lakes, NJ, USA) and analyzed using a BD FACSLyric™ flow cytometer and BD FACSuite v1.6 software (BD, Franklin Lakes, NJ, USA).

### 4.12. Statistical Analysis

For the statistical analysis of the results about characterization of grape seed extract, a one-way analysis of variance (ANOVA) was performed using SPSS (SPSS 19.0, Chicago, IL, USA) for all variables considered in the study. The least squares mean (LSM) values were separated using Fisher’s LSD test. All statistical tests of LSM were performed for a significance level of *p* < 0.05.

Statistical analyses and data visualization of flow cytometry results were performed using GraphPad Prism v10.5.0 (774). Results are presented as mean ± standard deviation (SD). Normality of data distribution was assessed using the Shapiro–Wilk and Kolmogorov–Smirnov tests to determine the appropriate statistical test (parametric or non-parametric) for comparisons. For normally distributed data, Student’s paired *t*-test was applied, while the Wilcoxon matched-pairs signed rank test was used for non-normally distributed data. A significance threshold of *p* ≤ 0.05 was considered for all tests.

## 5. Conclusions

The chemical composition and antioxidant capacity of Syrah grape seed extract were investigated. HPLC analysis showed that Syrah grape seed extracts were rich in monomeric flavan-3-ols: (+)-catechin, (−)-epicatechin, dimers: procyanidins B1, B2, B3 and trimer: procyanidin C1. Syrah grape seed extract was shown to have a high antioxidant capacity. GSE was found to exhibit immunomodulatory properties. The results obtained demonstrated a strong effect of GSE on Th1 cells, leading to an increased percentage of cells expressing HLA-DR alone and those co-expressing HLA-DR and CD25 in both study groups (healthy donors and SLE patients), with a more pronounced effect in PBMC from SLE patients. This study contributes to the elucidation of the mechanisms by which polyphenols in GSE influence immune function and provides valuable information about their regulatory potential in systemic lupus erythematosus and other autoimmune diseases.

## Figures and Tables

**Table 1:** Total phenolic content (TPC), total flavonoids (TF), procyanidins (PC) values of 1 g dry weight (dw) Syrah grape seed extract, stored at 23 °C for 6 months.

Polyphenolic Compounds	Liquid Extract		Dried Extract at 40 °C		Lyophilized Extract	
	0 Month	6 Months	0 Month	6 Months	0 Month	6 Months
TPC, mg GAE/g dw	226.22 ± 3.65 ^cA^	208.80 ± 4.6 ^cB^	237.21 ± 3.92 ^bA^	233.65 ± 4.2 ^bB^	240.06 ± 4.12 ^aA^	235.74 ± 2.7 ^aB^
TF, mg QE/g dw	150.27 ± 2.10 ^cA^	138.99 ± 3.8 ^cB^	161.81 ± 1.91 ^bA^	159.06 ± 3.5 ^bB^	171.36 ± 2.32 ^aA^	168.79 ± 3.7 ^aB^
PC, mg CE/g dw	119.88 ± 1.37 ^cA^	110.29 ± 2.8 ^cB^	130.74 ± 1.39 ^bA^	129.04 ± 2.9 ^bB^	139.14 ± 1.42 ^aA^	136.77 ± 4.5 ^aB^

GAE—gallic acid equivalent; QE—quercetin equivalent; CE—catechin equivalent. Small letters indicate statistically significant differences (*p* < 0.05) between the samples. The capital letters indicate statistically significant differences (*p* < 0.05) between stored condition (0 and 6 mounts). Values within the same column with different letters are significantly different (*p* < 0.05) according to Fisher’s LSD test.

**Table 2:** Antioxidant capacity of Syrah grape seed extract, stored at 23 °C for 6 months.

AO Assay	Liquid Extract		Dried Extract at 40 °C		Lyophilized Extract	
	0 Month	6 Months	0 Month	6 Months	0 Month	6 Months
ABTS, %	86.29 ± 0.54 ^cA^	79.29 ± 0.72 ^cB^	86.83 ± 0.62 ^bA^	85.23 ± 0.48 ^bB^	87.45 ± 0.65 ^aA^	86.35 ± 0.56 ^aB^
DPPH, %	80.12 ± 0.32 ^cA^	74.51 ± 0.67 ^cB^	80.72 ± 0.35 ^bA^	79.22 ± 0.63 ^bB^	82.35 ± 0.42 ^aA^	82.23 ± 0.47 ^aB^

Small letters indicate statistically significant differences (*p* < 0.05) between the samples. The capital letters indicate statistically significant differences (*p* < 0.05) between stored condition (0 and 6 mounts). Values within the same column with different letters are significantly different (*p* < 0.05) according to Fisher’s LSD test.

**Table 3:** Polyphenols in grape seed extracts of Syrah (*n* = 5, mean ± standard deviation).

Polyphenols	Retention Time, min	Absorbance, mAU	mg/g *
gallic acid	5.990 ± 0.11	20.64 ± 0.30	1.09 ± 0.22
procyanidin B1	14.120 ± 0.19	16.24 ± 0.24	11.88 ± 0.89
procyanidin B3	17.273 ± 0.25	7.00 ± 0.09	0.59 ± 0.06
(+)-catechin	19.300 ± 0.23	50.58 ± 0.55	22.6 ± 1.09
procyanidin B2	24.110 ± 0.30	15.80 ± 0.32	6.54 ± 0.59
(−)-epicatechin	27.127 ± 0.34	22.34 ± 0.54	10.15 ± 0.91
(−)-epigallocatechin gallate	28.480 ± 0.36	3.77 ± 0.03	0.25 ± 0.03
procyanidin C1	34.360 ± 0.31	11.95 ± 0.18	1.17 ± 0.88

* mg/g, expressed by dry weight.

**Table 4:** Characteristics of SLE patients. ANA—anti-nuclear antibodies; APLA—anti-phospholipid antibodies.

No. SLE Patient	Age	Sex	Disease Duration (Years)	Clinical Parameters Domain	Immunological Parameters	Therapy—4 mg Methylprednisolone Equivalent/Other
1.	54	M	25	Renal Mucocutaneous Musculoskeletal Serosal Hematologic	ANA, anti-dsDNA Abs, APLA	1t/-
2.	30	F	1	Renal Musculoskeletal Serosal Neuropsychiatric	ANA, anti-dsDNA Abs	2t/hydroxychloroquine (2 × 1t)
3.	43	M	22	Renal Mucocutaneous Musculoskeletal Serosal Hematologic	ANA, anti-dsDNA Abs APLA	2t/-
4.	78	F	2	Renal Hematologic	ANA, anti-dsDNA Abs	1t/hydroxychloroquine (2 × 1t)
5.	75	M	3	Renal Musculoskeletal Hematologic	ANA, anti-dsDNA Abs	1t/-
6.	43	F	1	Renal Musculoskeletal Serosal	ANA, anti-dsDNA Abs	2t/-
7.	48	F	5	Renal Mucocutaneous	ANA, anti-dsDNA Abs	1t/-
8.	45	M	4	Renal Musculoskeletal Serosal	ANA, anti-dsDNA Abs, APLA	2t/-
9.	67	M	1	Renal Mucocutaneous Musculoskeletal	ANA	1t/-
10.	34	F	2	Renal Mucocutaneous Hematologic	ANA	-/-
11.	44	F	3	Renal Musculoskeletal	ANA, anti-dsDNA Abs	1t/hydroxychloroquine (2 × 1t)
12.	58	F	1	Renal Musculoskeletal	ANA, anti-dsDNA Abs	-/hydroxychloroquine (2 × 1t)

## Data Availability

The original contributions presented in this study are included in the article. Further inquiries can be directed to the corresponding author.

## Abbreviations

The following abbreviations are used in this manuscript:

ABTS	2,2′-azino-bis (3-ethylbenzothiazoline-6-sulfonic acid)
ADs	autoimmune diseases
AO	antioxidant
DI H_2_O	deionized water
DPPH	2,2-diphenyl-1-picrylhydrazyl
dw	dry weight
GAE	gallic acid equivalent
GSE	grape seed extract
NK cells	natural killer cells
PBMC	peripheral blood mononuclear cells
PC	procyanidins
QE	quercetin equivalent
RP-HPLC	reverse phase high-performance liquid chromatography
SLE	systemic lupus erythematosus
TF	total flavonoids
TPC	total phenol content
